# Characterization of the complete chloroplast genome of *Murraya exotica* (Rutaceae) from Yunnan Province, China

**DOI:** 10.1080/23802359.2021.1942267

**Published:** 2021-07-09

**Authors:** Ning Zhou, Ai-Gen Fu

**Affiliations:** Chinese Education Ministry’s Key Laboratory of Western Resources and Modern Biotechnology, Key Laboratory of Biotechnology Shaanxi Province, College of Life Sciences, Northwest University, Xi’an, China

**Keywords:** Chloroplast genome, *Murraya exotica*, phylogenetic tree

## Abstract

*Murraya exotica* L. (Rutaceae) has important horticultural and medicinal values. Here, we reported the complete chloroplast (cp) genome of *M. exotica* using the next-generation sequencing method. The cp genome is 160,179 bp in length, including a large single-copy region (LSC, 87,726 bp), a small single-copy region (SSC, 18,465 bp), and a pair of inverted repeats (IR) regions 26,994 bp. A maximum-likelihood phylogenomic analysis showed that *M. exotica* was sister to *Murraya paniculate*. These findings will provide useful information for further investigation of cp genome evolution in *Murraya*.

*Murraya exotica* (Rutaceae) is a perennial tree widely cultivated in tropical and subtropical regions (Zhang and Hartley 2008). It is an important ornamental plant and medicine used for treating fever, cough, infectious wounds, and eliminating pain from injury and trauma (Forkuo et al. 2020). Previous studies have shown that many types of secondary coumarin compounds that occur in *M. exotica* are widely used in the medical, spice, and seasoning industries. The chloroplast (cp) genome can be used as a tool for phylogenetic analysis, and it can be used for other usages such as species identification (Li et al. 2015) or highly variable regions selection (Cui et al. 2020). In the present study, we assembled the complete cp genome of *M. exotica* to provide genomic and genetic sources for further research.

The fresh leaves of *M. exotica* were collected from Xishuangbanna Tropical Botanical Garden (E101°16′34.51′′, N21°55′10.87′′), Yunnan Province, China. The voucher specimen was deposited in the herbarium of Kunming Institute of Botany (KUN), Chinese Academy of Sciences (KUN1513863, http://www.kun.ac.cn/, dengtao@mail.kib.ac.cn). The genomic DNA was extracted following the modified CTAB method from leaf tissue (Doyle and Doyle 1987). Genome sequencing was performed on the Illumina HiSeq Platform (Illumina, San Diego, CA) at Genepioneer Biotechnologies Inc., Nanjing, China, and 6.9 GB of sequence data were generated. The low-quality reads and adapters were removed using CLC Genomics Workbench version 7.5 software (CLC Bio, Aarhus, Denmark) and the resulting high-quality reads were assembled via SPAdes (Bankevich et al. 2012). The assembled genome was annotated using Getorganelle (Jin et al. 2020). The cp genome was annotated using Geneious v10.2(Kearse et al. 2012). The annotated complete cp genome of *M. exotica* was deposited in GenBank (Genbank accession number: MW722359).

The cp genome of *M. exotica* was 160,179 bp in length, including a large single-copy region (LSC, 87,726 bp), a small single-copy region (SSC, 18,465 bp), and a pair of inverted repeats (IR) regions of 26,994 bp. The overall GC content was 38.6%, with the LSC, SSC, and IR regions being 37.10%, 34.80%, and 43.00%, respectively.

The phylogenetic analysis was performed using the complete cp genome of *M. exotica* and other species classified in the family Rutaceae and two species of *Meliaceae* designated as outgroups. The alignment was conducted using MAFFT (Katoh and Standley 2013). The phylogenetic tree was built using MEGA X (Kumar et al. 2018) with 1,000 bootstrap replicates and the Tamura–Nei model (Tamura and Nei 1993). *Murraya exotica* was sister to *Murraya paniculata*, especially, these two species and *Atalantia kwangtungensis* belonged to one clade (Figure 1). Therefore, the genus *Murraya* is not a monophyletic taxon, the result support previous systematic findings (Samuel et al. 2001; Groppo et al. 2008), and *Atalantia*, *Murraya*, *Glycosmis*, and *Clausena* in Aurantioideae formed monophyly (Wang et al. 2021). This baseline genomic study lays the foundation for future population genomic studies investigations, phylogenetic analyses, and genetic engineering studies of *M. exotica.*

**Figure 1.**
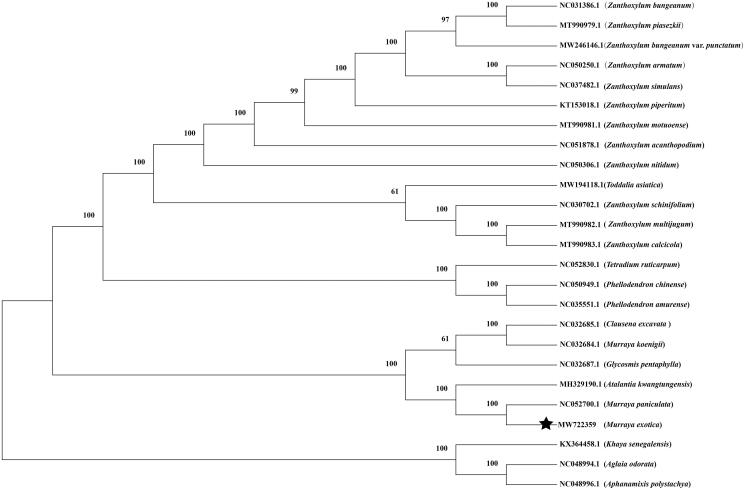
The maximum-likelihood phylogenetic tree constructed from 25 species chloroplast genomes. Numbers below or above branches are assessed by ML bootstrap.

## Data Availability

The genome sequence data that support the findings of this study are openly available in GenBank of NCBI at https://www.ncbi.nlm.nih.gov under the accession No. MW722359. The associated BioProject, SRA, and Bio-Sample numbers are PRJNA732890, SUB9737033, and SAMN19349668 respectively.
